# DETERIORATING HEALTH AMONG OLDER ADULTS AND CORTISOL: LONGITUDINAL EVIDENCE FROM THE MIDUS STUDY

**DOI:** 10.1093/geroni/igz038.955

**Published:** 2019-11-08

**Authors:** Julie Ober Allen

**Affiliations:** University of Michigan, Ann Arbor, Michigan, United States

## Abstract

Various mental and physical health conditions common among older adults have been linked to cortisol dysregulation (i.e., blunting of daily cortisol patterns) in predominantly cross-sectional studies. Researchers have suggested that cortisol dysregulation interferes with regulatory functions throughout the body and brain, disrupting multiple biological systems, and contributing to the development or progression of negative health outcomes over time. Prospective studies are needed to investigate the causal direction of cortisol dysregulation and poor health outcomes. This study examined whether diurnal cortisol patterns predicted subsequent health deterioration using longitudinal data from the National Survey of Midlife in the US (MIDUS). Analysis was restricted to 1,336 participants who provided salivary cortisol (4 samples/day for 4 days) and health data in MIDUS II (2004-2009) and updated health data in MIDUS III (2013-2014) (mean age=56, 45% male, 94% White). We simultaneous modeled multiple measures of diurnal cortisol patterns and their relationships to changes in mental (depressive symptomology) and physical (self-rated physical health, functional limitations, and number of new chronic health conditions) health from MIDUS II to III. All indicators of physical health deterioration were associated with cortisol, though not all measures demonstrated relationships in the expected direction. Mental health change over time was unrelated to cortisol. Older age was also associated with increased functional limitations and more new chronic conditions but improvements in mental health over time. Findings suggest that diurnal cortisol patterns contribute to physical health deterioration over time, independent of age-related decline, but not mental health changes in later life.

